# Effects of transcranial photobiomodulation on performance and cardiovascular responses in trained cyclists

**DOI:** 10.3389/fphys.2026.1766916

**Published:** 2026-03-16

**Authors:** Tommaso Arrighi, Andrea Meloni, Giulia Zaccaria, Annalisa Prato, Antonio La Torre, Livio Luzi, Roberto Codella, Luca Filipas

**Affiliations:** 1 Department of Biomedical Sciences for Health, Università degli Studi di Milano, Milan, Italy; 2 Department of Economic, Psychological, Communication, Education and Movement Sciences, Università degli Studi Niccolò Cusano, Rome, Italy; 3 Department of Endocrinology, Nutrition and Metabolic Diseases, IRCCS MultiMedica, Milan, Italy; 4 TotalEnergies Pro Cycling Team, Essarts-enBocage, France

**Keywords:** brain stimulation, cycling performance, photobiomodulation, prefrontal cortex, time trial

## Abstract

**Background:**

Transcranial photobiomodulation (PBM) has been proposed to enhance prefrontal cortex (PFC) oxygenation and modulate central mechanisms of fatigue, potentially improving endurance performance. This study investigated the acute effects of PBM on cycling time-trial (TT) performance in well-trained cyclists.

**Methods:**

In a randomized, double-blind, crossover design, 18 trained cyclists completed two experimental conditions (PBM and SHAM) prior to a constant-load (CL) test at 5% above the first lactate threshold (LT1) and a 25-min self-paced TT on their own bicycles mounted on an ergometer.

**Results:**

No significant condition × time interactions were found for heart rate, blood lactate, ratings of perceived exertion (RPE), or power-related ratios during either the constant-load or TT trials (p > 0.05).

**Conclusion:**

Acute transcranial PBM did not influence cycling performance or perceptual and physiological responses in trained athletes. These findings suggest that the applied PBM parameters may have been insufficient to elicit measurable cortical or performance effects. Future research should explore optimized stimulation parameters and chronic application protocols to clarify the potential ergogenic role of PBM in endurance exercise.

## Introduction

Photobiomodulation (PBM) is a cellular stimulation technique that uses red to near-infrared (NIR) light to modulate cellular activity (Gonzalez-Lima and Rojas, 2011). The most commonly used wavelengths range between 600 and 1100 nm, as they do not produce heat or cause tissue damage (Hamblin and Demidova, 2006).

Mitochondria play a central role in mediating the effects of PBM at the cellular level. Components of the mitochondrial respiratory chain have been identified as key absorbers of PBM. In particular, cytochrome c oxidase, myoglobin, and cytochrome b have been highlighted as principal photoacceptors (Eells et al., 2004; Karu et al., 2005). Additionally, enzymes such as superoxide dismutase, cytochrome c, and nitric oxide synthase contribute to PBM absorption within the red to near-infrared spectrum (Karu, 1999; Karu et al., 1996).

At the cellular level, the effects of PBM are diverse (Gonzalez-Lima and Rojas, 2011). These effects can be attributed to photochemical changes within photoacceptors following light stimulation. Specifically, atypical reactivity in oxidation–reduction reactions within the mitochondrial respiratory chain has been observed, leading to increased ATP synthesis, free-radical generation, and transient localized heating within the cell (Hamblin and Demidova, 2006; Karu, 1999). This triggers a cascade of biochemical reactions, activating second messengers that modulate enzymatic activity and gene expression via newly engaged cellular signaling pathways, ultimately altering cellular function (Hamblin et al., 2011; Karu, 2010).

The literature indicates that PBM effects vary by tissue type. In muscle tissue, PBM has been associated with increased oxygen demand (Lanferdini et al., 2023; Pruitt et al., 2022), promoting greater aerobic efficiency and improved performance in exhaustion tests (Dutra et al., 2022). Positive effects have also been reported on muscle repair, including reduced inflammation, modulation of growth factors and myogenic regulatory factors, and increased angiogenesis (Alves et al., 2014).

In nervous tissue, beyond the metabolic benefits observed in muscle, PBM has shown neuroprotective effects, attributed to increased antioxidant and anti-apoptotic defenses that reduce oxidative stress and pro-apoptotic signaling in neurons (Liang et al., 2006; 2008). Transcranial PBM has also been reported to enhance cognitive abilities in healthy individuals (Nairuz et al., 2024; Tian et al., 2016) and shows potential utility in neurodegenerative conditions (Chan et al., 2022; Chao, 2019; Chao et al., 2020; O’Donnell et al., 2023). The underlying mechanism appears to involve increased cerebral blood flow and enhanced hemoglobin oxygenation, leading to greater metabolic activity and activation of irradiated brain areas.

The prefrontal cortex (PFC) plays a central role in integrating cognitive and emotional information during exercise. As the brain continuously processes bodily signals related to effort, pain, arousal, and affective state, the PFC contributes to attentional regulation, inhibitory control, and the interpretation of interoceptive cues, all factors known to influence endurance performance (Robertson and Marino, 2016; Angius et al., 2019). Modulating activity within these regions has been shown to alter affective responses to exercise, potentially improving the ability to sustain high-intensity efforts over time (De Wachter et al., 2021).

In the context of physical exercise, the time trial (TT) is commonly employed as a timed test to stimulate endurance performance. Its validity and repeatability in trained cyclists have been confirmed by multiple studies (Funnell et al., 2023; Smith et al., 2001; Thomas et al., 2012). Several investigations have specifically assessed the effect of PFC-targeted interventions. For instance, Pollastri et al. (2021) reported performance improvements in healthy athletes following anodal high-definition transcranial direct current stimulation (HD-tDCS), while similar enhancements were observed by Filipas et al. (2022) in athletes with type I diabetes. Furthermore, significant gains in TT performance have been documented after brain endurance training protocols (Staiano et al., 2023). Complementing these findings, Santos-Concejero et al. (2015) observed increased PFC oxygenation and blood flow during the early stages of a 5-km TT in high-level Kenyan long-distance runners, suggesting a link between PFC engagement, effort tolerance, and endurance performance. Moreover, at least one study has reported the absence of positive effects on PFC-related outcomes following a single session of transcranial-PBM stimulation (Khoury et al., 2021). However, PBM mechanisms are closely linked to mitochondrial activity, oxygen utilization, and metabolic demand, which are substantially elevated during exercise compared to rest. Therefore, the absence of detectable effects at rest does not necessarily preclude potential benefits under the heightened physiological and metabolic stress of endurance exercise, where cerebral oxygenation, neuronal activation, and regulatory demands are markedly increased. Unfortunately, to date, no studies have investigated the effectiveness of transcranial PBM during TT performance.

Collectively, these results highlight the functional relevance of PFC activity in endurance tasks and support the notion that modulating prefrontal mechanisms, whether through neurostimulation, cognitive training, or other interventions, can influence effort perception, pacing decisions, and overall athletic performance.

Considering the evidence above and the current lack of studies investigating the effectiveness of transcranial PBM in endurance exercise, the aim of the present study was to evaluate the potential acute positive effects of transcranial PBM in trained cyclists from both physiological and performance perspectives, using a constant-load (CL) test and a TT.

## Materials and methods

### Participants

A total of 20 male trained amateur road cyclists volunteered to participate in this study. The sample size estimation was based on effect sizes reported in previous studies employing similar protocols and outcome measures. Specifically, the estimation was informed by prior studies conducted in our laboratory to ensure that participants would reach exhaustion within the planned experimental duration. Two participants were excluded from the final analysis due to non-compliance with days between testing sessions instructions, therefore 18 participants were eligible for the study. Based on each participant’s training history (6 [2] years of practice) and competitive level, they can be classified as “Trained/Developmental” according to guidelines proposed by McKay and colleagues (McKay et al., 2022). Each athlete was informed of the procedures and risks before giving a written informed consent to participate in the study. The study design and procedures were approved by the local research ethics committee of the Università degli Studi di Milano (n° 52/20) and followed the ethical principles for medical research involving human participants set by the World Medical Association Declaration of Helsinki.

### Experimental design

**FIGURE 1:**
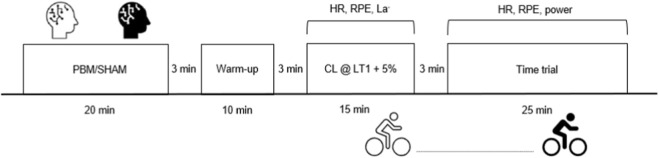
Schematic overview of the experimental design. Participants completed three laboratory visits: Visit one involved baseline lactate-threshold testing and familiarization. Visits 2 and 3, conducted in randomized, double-blind, counterbalanced order, included either PBM or SHAM stimulation followed by a warm-up, a 15-min constant-load (CL) test, and a 25-min self-paced time trial (TT). HR = heart rate; RPE = rating of perceived exertion; La^−^ = blood lactate.

The participants visited the laboratory on three different occasions. During the first visit, they initially performed a cycling lactate threshold test to establish power output at first (LT1) and second (LT2) lactate threshold, then they were familiarized with the constant load and the TT to be performed on sessions 2 and 3. On second and third visits (Figure 1), in a double blind and counterbalanced order, participants underwent either the experimental treatment (PBM) or SHAM, with both interventions lasting 20 min. Three minutes after the end of the treatment, participants carried out 10 min warm up at 80% of LT1. Three minutes after the end of the warmup they performed 15 min constant load trial (CL) at the power output of LT1 + 5%. Then, they rested for 3 min seated on the bike and subsequently performed a simulated 25-min TT. Participants carried out all cycling test on the frame of their own bike, fitted on Direto X-RT ergometer (Elite, Fontaniva, Italy). Each subject completed all sessions within a maximum of 2 weeks. The day before each visit they performed a standardized training session (volume: < 3 h, intensity: < LT1). Compliance with these guidelines has been verified before each experimental session. The sessions were completed at the same time of the day (±2 h). The participants were asked to maintain the same diet on the day of the tests and were asked not to consume caffeine, in order to minimize external influences on the trials.

### Lactate threshold test

The test protocol was intermittent and incremental. It began with a 10-min warm-up, at an intensity between 120 W and 150 W, depending on athlete weight and estimated Functional Threshold Power (FTP) (Mackey and Horner, 2021). This specific intensity also served as the intensity for the active recovery phases. The main part of the test consisted of 4-min work stages interspersed with 1-min active recovery intervals. The initial workload was individualized based on each athlete’s estimated FTP, defined as the highest power output that can be sustained for one hour. At each subsequent stage, power output was increased by 30 W. The test was terminated when blood lactate concentration exceeded 4 mmol/L. LT1 and LT2 were determined based on fixed blood lactate thresholds, corresponding respectively to an onset of blood lactate accumulation (OBLA) of 2.0 mmol/L and 4.0 mmol/L (Heck et al., 1985).

### Constant load and time trial

The CL consisted of performing 15 min at LT1 power output +5% (246,67 [32,18] W) at a freely chosen cadence. After 3 min of passive rest sitting on the bike, they performed a simulated 25-min TT, with the aim to maintain the maximal power output sustainable. The TT began with the standard virtual gear, and then the participants were free to shift it. The only feedback provided during TT was the time elapsed from the beginning. The participants were allowed to drink water *ad libitum*, while no verbal encouragement was provided. During both the CL and TT, the ergometer recorded the power output. For data analysis, we averaged these parameters in splits of 5 min in length. The participants were blinded to their performance and physiological data until the end of the entire protocol.

### Physiological and perceptual measures

During visits 2 and 3, the heart rate (HR) and rate of perceived exertion (RPE) were measured every 5 min during both CL and TT. The heart rate was recorded as the average of the last 30 s before every time point, using a chest strap (Polar H10; Polar Electro Oy, Kempele, Finland) connected to the ergometer software (My E-Training; Elite, Fontaniva, Italy). The RPE was recorded using Borg 6 to 20 scale (Borg, 1998) to measure whole-body perceived exertion. The standardization procedures have been followed both during the instructions and the scale administration. During the CL blood samples were taken every 5 min from the ear lobe for the determination of blood lactate (La^-^) (Lactate Pro 2; Arkray Inc, Tokyo, Japan).

### Transcranial PBM procedures

Transcranial PBM was delivered using Neuro Gamma 3 device (Vielight Inc., Toronto, Ontario, Canada), for a duration of 20 min. It consists of 4 LEDs, each one emitting NIR light (810 nm), pulsating at 40 HZ, over a beam spot of 1 cm^2^: three with irradiance of 100 mW/cm^2^, and the fourth 75 mW/cm^2^. Moreover, the device includes an intranasal probe with one LED emitting pulsed NIR light with an irradiance of 25 mW/cm^2^ into the right nasal cavity. This device configuration, previously employed in multiple transcranial PBM studies (Chan et al., 2022; Chao et al., 2020; Johnson et al., 2024), is designed to target the PFC. In the SHAM condition the placement of the device was the same, but stimulation was not active. All participants tolerated PBM well, and no side effects were reported during or after the sessions.

### Statistical analysis

All data are presented as mean (SD). The assumptions of normality and sphericity were checked using the Shapiro-Wilk test and the Mauchly test, respectively. All the data showed normal distribution, while the Greenhouse-Geisser correction was used when sphericity was not met. A paired t-test was used to compare the overall mean values calculated over the entire duration of the test for HR during CL and power and HR during TT. A two-way repeated-measures analysis of variance (RM-ANOVA) was performed to analyse La^-^, HR, RPE, power/RPE and power/HR during CL; power, HR, RPE, power/RPE and power/HR during the TT. Effect sizes for RM-ANOVA are reported as partial eta squared (η^2^_p_), using the small (<0.13), medium (0.13 - 0.25) and large (>0.25) interpretation for effect size (Bakeman, 2005), while effect sizes for pairwise comparison were calculated using Cohen’s d and are considered to be either trivial (<0.20), small (0.21 - 0.60), moderate (0.61 - 1.20), large (1.21 - 2.00), or very large (>2.00) (Hopkins et al., 2009). For all analyses, a p value less than 0.05 was considered statistically significant. The data analysis was performed using the GraphPad Prism software (GraphPad Software, Version 11, San Diego, CA, USA), and data collection was performed using Excel version 16.32 for Mac (Microsoft, Redmond, WA, USA).

## Results

### Participants

Descriptive characteristics and lactate threshold test results of cyclists are presented in Table 1.

**TABLE 1:** Characteristics of the study participants (n = 18).

Variable	Mean ± SD
Age (years)	29.5 ± 4.1
Weight (kg)	71.8 ± 7.0
Height (cm)	181.0 ± 4.9
LT1 (W)	234.2 ± 26.6
LT1 (W/kg)	3.28 ± 0.44
LT2 (W)	290.9 ± 39.8
LT2 (W/kg)	4.06 ± 0.44

Abbreviations: LT1, first lactate threshold; LT2, second lactate threshold.

### Constant load

**FIGURE 2:**
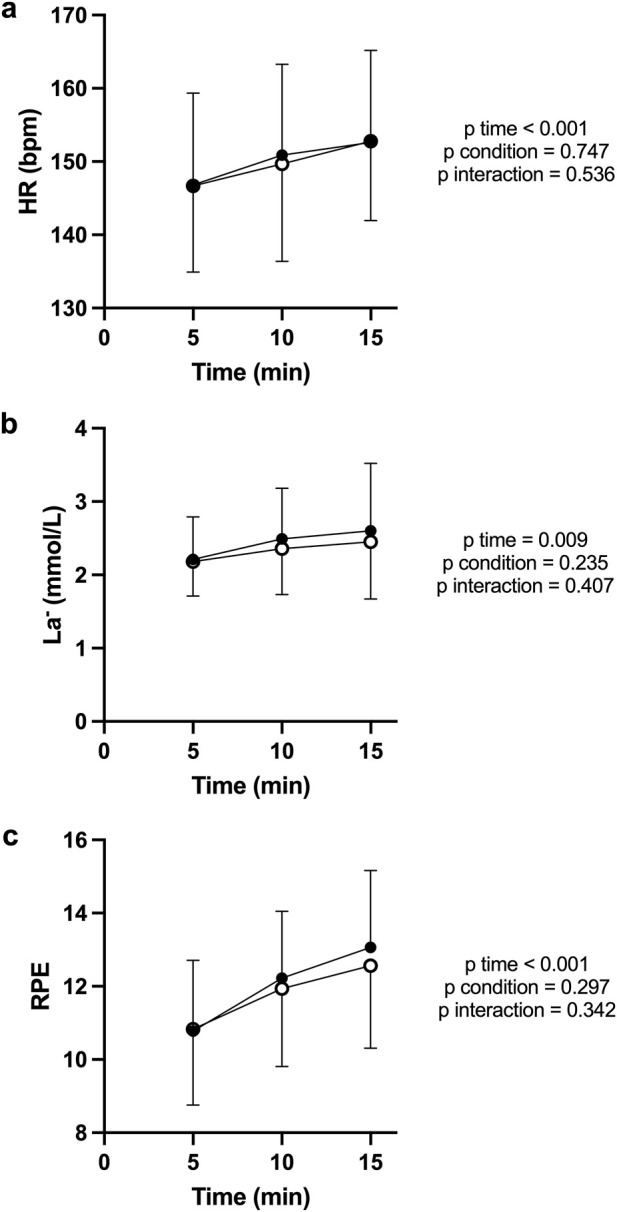
Heart rate **(a)**, blood lactate concentration **(b)**, and perceived exertion **(c)** during the CL test in PBM (filled circles) and SHAM (open circles) conditions. Values are mean ± SD at 5-min intervals. HR and La^−^ increased progressively across time, with no significant differences between conditions. RPE rose steadily, reflecting the expected perceptual progression with sustained effort.

Overall mean HR (PBM: 147.06 [11.59] bpm, SHAM: 147.33 [11.60] bpm) showed no difference between conditions (p = 0.793; d = 0.06). HR increased progressively over time in both conditions (Figure 2a), confirming a typical physiological rise with sustained effort (F (2, 34) = 39.32; p < 0.001; η^2^_p_ = 0.72). No main effect of condition (F (1, 17) = 0.108; p = 0.747; η^2^_p_ = 0.02) and no condition × time interaction were observed (F (2, 34) = 0.635; p = 0.536; η^2^_p_ = 0.04).

Lactate concentration (Figure 2b) rose gradually throughout the CL, consistent with a moderate steady-state load (F (2, 34) = 5.458; p = 0.009; η^2^_p_ = 0.58), with no significant differences between PBM and SHAM (F (1, 17) = 1.516; p = 0.235; η^2^_p_ = 0.17) and no interaction condition x time (F (2, 34) = 0.924; P = 0.407; η^2^_p_ = 0.05).

RPE increased systematically across time (F (2, 34) = 24.23; p < 0.001; η^2^_p_ = 0.77), mirroring HR and La^−^ responses, but did not differ between conditions (F (1, 17) = 1.160; p = 0.297 η^2^_p_ = 0.07) and no interaction condition x time (F (2, 34) = 1.108; P = 0.342; η^2^_p_ = 0.06).

**FIGURE 3:**
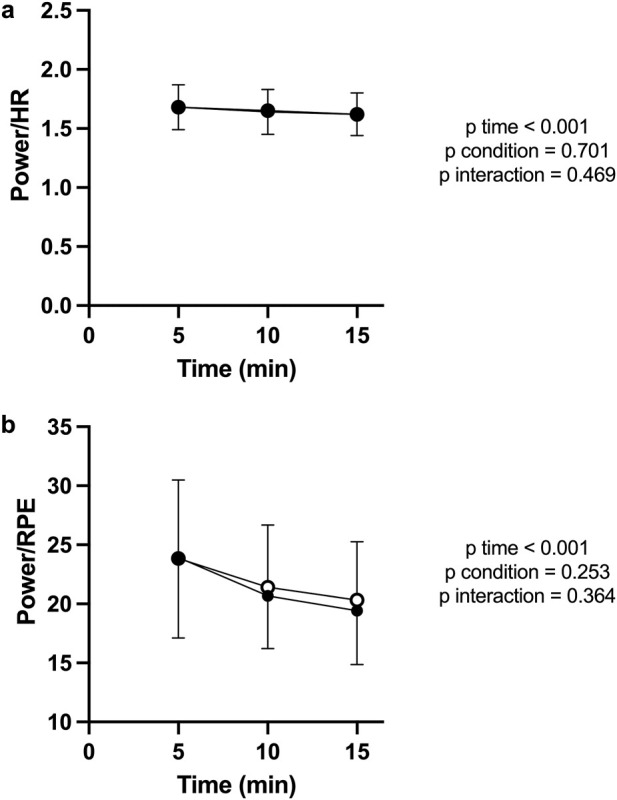
Power/HR ratio **(a)** and Power/RPE ratio **(b)** during the CL test for PBM (filled circles) and SHAM (open circles). Both indices declined significantly over time, consistent with increasing physiological strain. Curves overlapped closely between PBM and SHAM, indicating no treatment effect.

For power/HR ratio (Figure 3a) there was a significant main effect of time (F (2, 34) = 38.01; P < 0.001; η^2^_p_ = 0.74), while no main effect of condition (F (1, 17) = 0.132; P = 0.701; η^2^_p_ = 0.02), and no interaction condition x time (F (2, 34) = 0.774; P = 0.469; η^2^_p_ = 0.04).

For power/RPE ratio (Figure 3b) there was a significant main effect of time (F (2, 34) = 19.70; P < 0.001; η^2^_p_ = 0.79), while no main effect of condition (F (1, 17) = 1.398; P = 0.253; η^2^_p_ = 0.08), and no interaction condition x time (F (2, 34) = 1.040; P = 0.364; η^2^_p_ = 0.06).

### Time trial performance

**FIGURE 4:**
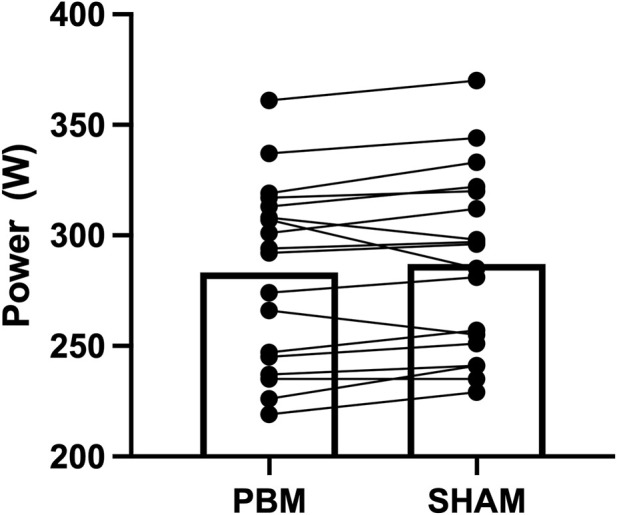
Overall mean power output during the 25-min TT under PBM and SHAM conditions.

**FIGURE 5:**
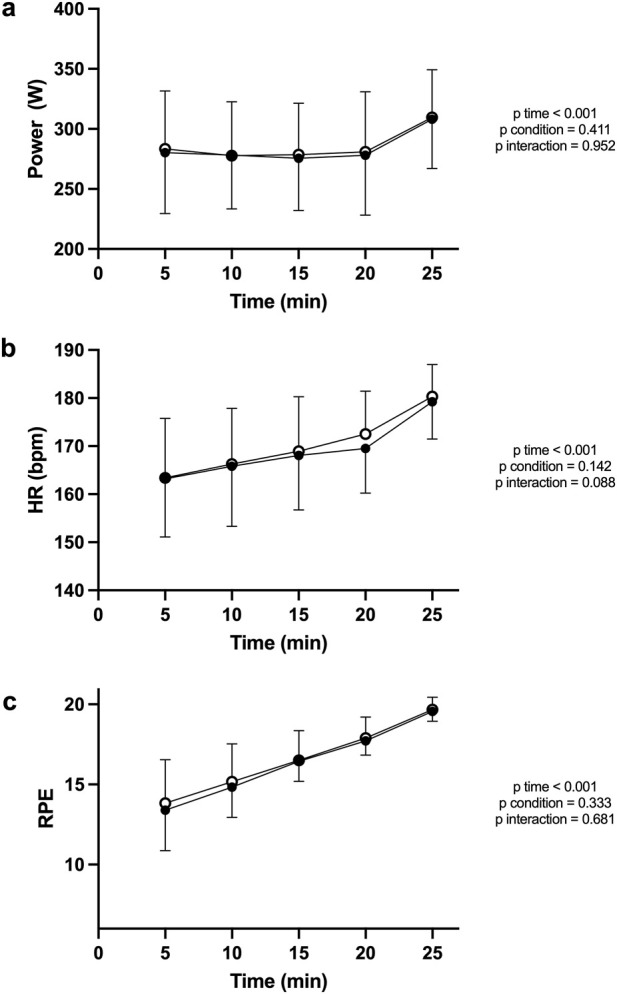
Power **(a)**, heart rate **(b)** and perceived exertion **(c)** responses during the 25-min TT for PBM (filled circles) and SHAM (open circles). Values are mean ± SD at 5-min intervals. For power a small, non-significant trend toward higher early PBM power was observed, converging with SHAM thereafter. Both HR and RPE increased progressively across time (main effect of time, p < 0.001) with no condition or interaction effects.

Overall mean power during TT was 283.22 [41.27] W in the PBM condition and 287.06 [41.34] W in the SHAM condition, with no significant difference between them (p = 0.105; d = 0.40; Figure 4). Power output (Figure 5a) declined significantly over time (F (4, 68) = 8.288; p < 0.001; η^2^_p_ = 0.76) with no effect of condition (F (1, 17) = 0.712; p = 0.411) or interaction (F (4, 68) = 0.172; p = 0.952; η^2^_p_ = 0.01).

Overall mean HR was 165.89 [10.36] bpm in the PBM condition and 167.17 [10.12] bpm in the SHAM condition, with no significant difference between them (P = 0.361; d = 0.22).

For HR (Figure 5b) there was a significant main effect of time (F (4, 68) = 40.75; p < 0.001; η^2^p = 0.94), but no effect of condition (F (1, 17) = 0.785; P = 0.142; η^2^_p_ = 0.14), and no interaction condition x time (F (4, 68) = 2.121; P = 0.088; η^2^_p_ = 0.11).

RPE (Figure 5c) followed a similar pattern (F (4, 68) = 81.27; p < 0.001; η^2^p = 0.97), did not vary by condition (F (1, 17) = 0.945; P = 0.333; η^2^_p_ = 0.07), with no interaction condition x time (F (4, 68) = 0.576; P = 0.681; η^2^_p_ = 0.03).

**FIGURE 6:**
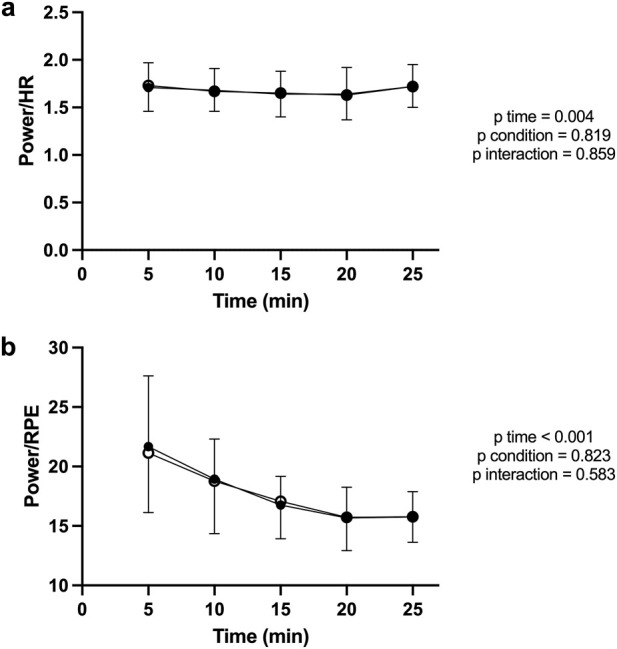
Power/HR ratio **(a)** and Power/RPE ratio **(b)** during the TT. Both ratios declined significantly with time, indicating fatigue-related efficiency loss. No significant differences were found between PBM and SHAM.

For power/HR ratio (Figure 6a) there was a significant main effect of time (F (4, 68) = 4.244; P = 0.004; η^2^_p_ = 0.54), while no main effect of condition (F (1, 17) = 0.054; P = 0.819; η^2^_p_ < 0.01), and no interaction condition x time (F (4, 68) = 0.328; P = 0.859; η^2^_p_ = 0.02).

For power/RPE ratio (Figure 6b) there was a significant main effect of time (F (4, 68) = 19.66; P < 0.001; η^2^_p_ = 0.91), while no main effect of condition (F (1, 17) = 0.052; P = 0.823; η^2^_p_ < 0.01), and no interaction condition x time (F (4, 68) = 0.712; P = 0.583; η^2^_p_ = 0.04).

Overall, both the CL and TT produced internally consistent physiological responses, but PBM did not alter performance, cardiovascular strain, or perceived effort.

## Discussion

The present study evaluated the acute effects of transcranial PBM on endurance performance in trained cyclists. To the best of our knowledge, this is the first investigation examining the impact of this specific modality of brain stimulation on physical performance. The results revealed no significant improvements in mean power output, HR, RPE, power/HR ratio or power/RPE ratio during either the submaximal CL or self-paced TT. Only significant effects of time were observed across variables, confirming the expected physiological drift during sustained cycling but no influence of PBM. Visual inspection of Figures 2–6 further confirmed that PBM and SHAM trajectories were nearly superimposed, supporting the statistical findings.

Taken together, these findings indicate that the acute application of transcranial PBM, as implemented in this study, did not elicit measurable changes in performance, cardiovascular responses, perceived exertion, or efficiency-related indices during either a self-paced TT or a submaximal CL test. Prior investigations using HD-tDCS have reported improvements in TT performance and more favorable relationships between external and internal load (Filipas et al., 2022; Pollastri et al., 2021), likely due to the used technology, which directly modulates cortical excitability via electrical currents, while PBM relies on optical energy whose penetration through scalp and skull is limited.

The PFC is a central hub in the integration of cognitive, affective, and interoceptive signals during exercise. Its activation supports attentional regulation, inhibitory control, pacing decisions, and the perception of effort and fatigue. Enhanced PFC engagement has been associated with improved endurance performance, as evidenced by studies showing increased prefrontal oxygenation during the early stages of a 5-km time trial in elite runners (Santos-Concejero et al., 2015) and during static muscular endurance tasks, where self-regulation strategies modulated PFC oxygenation (Wolff et al., 2018). These findings underscore that not only is the PFC crucial for integrating physiological and psychological signals during exercise, but the way in which individuals regulate effort cognitively can directly influence PFC activation and, potentially, performance outcomes. Supporting this notion, recent evidence targeting the PFC with HD-tDCS in healthy adults has examined its effect on psychophysiological responses and brain oxygenation during exercise. Despite theoretical expectations that anodal HD-tDCS could reduce perceived effort by modulating prefrontal networks, a study reported no significant changes in cerebral oxygenation or psychophysiological variables compared with sham (Machado et al., 2025). These findings highlight that single-session prefrontal neuromodulation may not reliably translate into measurable changes in cerebral hemodynamics or endurance performance, particularly in populations with already elevated baseline PFC activation, such as endurance-trained athletes (Santos-Concejero et al., 2015).

The Vielight device used here (810 nm, 40 Hz, ≤ 100 mW cm^-2^) likely resulted in sub-cortical irradiance levels insufficient to induce measurable neuromodulation within the PFC. Studies reporting acute increases in cerebral oxygenation after PBM (O’Donnell et al., 2023; Tian et al., 2016) generally employed higher irradiances or longer wavelengths (≥1064 nm) than used here, potentially explaining divergent results. Our findings align instead with those of (Khoury et al., 2021), who found no difference in brain activation after a single PBM session. Collectively, the evidence suggests that acute PBM may require higher dosimetry or repeated exposure to induce measurable cortical or performance effects.

In the end, peripheral PBM applications have consistently improved muscular bioenergetics through enhanced mitochondrial ATP production and reduced oxidative stress (Dutra et al., 2022; Pruitt et al., 2022). However, transcranial PBM may not exert such direct effects on muscle metabolism. The lack of changes in HR, lactate, and perceived exertion in our data supports the view that the stimulation did not meaningfully alter cortical regulation of pacing or central fatigue.

The main limitation of this study is the lack of concurrent neuroimaging (e.g., fNIRS or EEG) to confirm cortical engagement. Such measures would clarify whether stimulation reached the PFC and whether null behavioral results reflect insufficient penetration or genuine absence of effect. Additionally, only acute responses were assessed; chronic PBM protocols might elicit cumulative neuroplastic or hemodynamic adaptations not observable after one exposure.

Future studies should employ optimized wavelengths and irradiance levels validated for cortical penetration, combine PBM with neuroimaging to verify target engagement, and explore repeated-session paradigms. Investigating less trained populations may also be informative, as lower baseline cortical activation could make them more responsive to PBM.

Another important limitation is the absence of direct measures of executive function, decision-making, pacing strategy, or perceptual responses during exercise. Although the theoretical rationale links PFC modulation to these higher-order processes, no behavioral or cognitive assessments (e.g., inhibitory control tasks such as the Stroop test) were included to determine whether transcranial PBM influenced executive control mechanisms beyond global performance outcomes. In summary, despite the theoretical rationale linking PFC oxygenation to endurance regulation, acute transcranial PBM failed to improve cycling performance or physiological responses in trained athletes. The data highlight the need for refined PBM protocols, mechanistic validation via neuroimaging, and exploration of chronic stimulation effects before firm conclusions can be drawn about its ergogenic potential.

## Data Availability

The original contributions presented in the study are included in the article/supplementary material, further inquiries can be directed to the corresponding author.
